# Coronavirus disease 2019 (COVID-19) infection prevention practices that exceed Centers for Disease Control and Prevention (CDC) guidance: Balancing extra caution against impediments to care

**DOI:** 10.1017/ice.2023.89

**Published:** 2023-12

**Authors:** Shruti K. Gohil, Edward Septimus, Kenneth E. Sands, Eunice Jackie Blanchard, Julia Moody, Annabelle de St. Maurice, Deborah Yokoe, Jennie Kwon, Jonathan Grein, Stuart Cohen, Daniel Uslan, Milind Vasudev, Amarah Mauricio, Shannon Mabalot, Micaela H. Coady, Selsebil Sljivo, Kimberly Smith, Brandon Carver, Russell Poland, Jonathan Perlin, Richard Platt, Susan S. Huang, for the Centers for Disease Control and Prevention Epicenters Program

**Affiliations:** 1 Epidemiology & Infection Prevention Program, University of California Irvine Health (UC Irvine Health), Irvine, California; 2 Division of Infectious Diseases, University of California, Irvine School of Medicine, Irvine, California; 3 Department of Population Medicine, Harvard Pilgrim Healthcare Institute, Harvard Medical School, Boston, Massachusetts; 4 HCA Healthcare, Nashville, Tennessee; 5 Division of Pediatric Infectious Diseases, David Geffen School of Medicine at University of California Los Angeles, Los Angeles, California; 6 Division of Infectious Diseases, University of California San Francisco, San Francisco, California; 7 Division of Infectious Diseases, Washington University School of Medicine, St. Louis, Missouri; 8 Division of Infectious Diseases, Cedars Sinai Medical Center, Los Angeles, California; 9 Division of Infectious Diseases, University of California Davis, Davis, California; 10 Division of Infectious Diseases, David Geffen School of Medicine at University of California Los Angeles, Los Angeles, California; 11 Infection Prevention, Sharp Metropolitan Medical Campus, San Diego, California

## Abstract

In a survey of infection prevention programs, leaders reported frequent clinical and infection prevention practice modifications to avoid coronavirus disease 2019 (COVID-19) exposure that exceeded national guidance. Future pandemic responses should emphasize balanced approaches to precautions, prioritize educational campaigns to manage safety concerns, and generate an evidence-base that can guide appropriate infection prevention practices.

Personal protective equipment (PPE) is used to protect healthcare personnel (HCP) and patients from exposure to transmissible diseases across a variety of healthcare activities. Infection prevention (IP) programs rely on public health guidance, clinical and epidemiologic evidence, and experience to limit infection transmission while assuring occupational safety with minimal disruption to patient care.

The arrival of severe acute respiratory coronavirus virus 2 (SARS-CoV-2) as a novel infectious pathogen has raised questions about the adequacy of PPE strategies to prevent transmission in healthcare settings. Early observations in China reported HCP infection rates 12-fold higher than in the community.^1^ Subsequent studies assessing HCP transmission in the setting of adequate PPE and IP protocols showed that acquisition of coronavirus disease 2019 (COVID-19) by HCP was predominantly due to community exposure.^2^ Nevertheless, early in the pandemic, fear and perceived risk was understandably high among HCP due to the severity of illness and lack of a vaccine. Presymptomatic transmissibility furthered concerns about acquiring COVID-19 during patient care.

Concerns about infection led many HCP to question the effectiveness of IP strategies deployed to protect them. We assessed the early pandemic experiences of hospitals balancing HCP protection through PPE use and delivery of timely, high-quality patient care.

## Methods:

We conducted a 32-question, structured survey of a convenience sample of hospital IP leaders recruited from 4 sources: (1) the Centers for Disease Control and Prevention Epicenters Program, (2) HCA Healthcare hospital system, (3) the University of California Health system, and (4) the California Metrics Group for Healthcare-Associated Infections. Surveys were emailed and received between May 4, 2020, and November 20, 2020. The survey closed November 30, 2020. Responses were restricted to 1 survey per hospital. Survey questions evaluated (1) HCP concerns leading to procedure avoidance or delays, (2) modifications in clinical or IP workflows, and (3) PPE-related occupational hazards. Data were aggregated across facilities. Percentages were calculated among respondents for each question. This research was exempt from human subjects review by the UC Irvine Institutional Review Board.

## Result

Of 130 programs receiving the survey, responses were received from IP program leaders at 53 US hospitals across 15 states (response rate, 41%). All hospitals provided ICU care and 29 (55%) were academic facilities. By size, 22 facilities (42%) had <200 beds, 14 (26%) had 200–400 beds, and 17 (32%) had >400 beds. Care services for immunocompromised patients were provided by 22 facilities (41%) and level 1 trauma care was provided by 11 facilities (21%). Overall, 40 facilities (75%) had experienced a COVID-19 surge by the time of survey completion.

Table 1 summarizes responses to questions regarding procedure avoidance or delays due to HCP concerns about COVID-19 risk. Delays or changes in care delivery resulting in longer hospital lengths of stay were reported by 42 (79%) of 53 facilities. Delays due to preprocedure COVID-19 testing were reported by 46 (87%), with 40 (75%) reporting unexpected cancellations. Also, 37 facilities (70%) reported increases in emergency department (ED) visits due to COVID-19 disruption of routine medical management of chronic conditions (eg, diabetic ketoacidosis or hypertensive urgency).

**Table 1.** Healthcare Personnel Concerns Leading to Procedure Avoidance or Delays^a^

Survey Questions^b^	Ever, No. (%)^b^ ^c^	A Few Times (1–2×), No. (%)^c^	Sometimes (3–5×), No. (%)^c^	Often (>5×)^c^	Never, No. (%)^c^
**Healthcare personnel concerns leading to procedure avoidance or delays**					
**A. How often have you heard about delays or changes in hospital care leading to longer hospital stays?**	42 (79)	11 (21)	13 (24)	18 (34)	11 (21)
**B. During the COVID-19 pandemic, how often did you hear about the following occurring due to healthcare personnel concerns about COVID-19 transmission:**					
Procedure delay due to request for preprocedural COVID-19 testing	46 (87)	20 (38)	7 (13)	19 (36)	7 (13)
Unexpected cancellation/delay of non-OR procedure (eg, bronchoscopy, IR, cardiac catheterization, TEE, EGD) (N=52)	40 (77)	17 (33)	11 (21)	12 (23)	12 (23)
Unexpected cancellation/delay in surgery requiring general anesthesia in OR (eg, CABG, vascular surgery, biopsy, ex-lap)	40 (75)	15 (28)	10 (19)	15 (28)	13 (25)
Early intubation instead of high flow nasal cannula or other noninvasive positive pressure ventilation	26 (49)	11 (21)	9 (17)	6 (11)	27 (51)
**Clinical or infection prevention workflow modifications due to HCP COVID-19 concerns**					
**C. How often did you encounter modifications in usual clinical or infection prevention workflows due to concerns about COVID-19 transmission?**					
Use of extra PPE affecting surgical procedure times (eg, double PPE, body suits/PAPRs requiring extra time for doffing and donning) (N=52)	37 (71)	15 (29)	6 (11)	16 (30)	15 (29)
Difficulty completing a procedure due to reduced visibility from face shields/goggles	40 (75)	9 (17)	12 (23)	19 (36)	13 (25)
Difficulty with a procedure due to double-gloving (eg, IV insertion, central line insertion, etc)	10 (19)	6 (11)	2 (4)	2 (4)	42 (79)
Avoidance of preoperative infection prevention protocols such as nasal decolonization due to theoretical impact to COVID-19 test accuracy. (N=52)	9 (17)	4 (7)	3 (6)	2 (4)	43 (83)
Request to allow time for air exchanges between patients (eg, in OR, ED)	51 (96)	8 (15)	15 (28)	28 (53)	2 (4)
Requests for or inquiries into changing OR from positive to negative air pressure (N=52)	33 (63)	14 (27)	9 (17)	10 (19)	19 (37)
Procedure modifications (eg, cauterization not allowed/discouraged due to AGP concern) (N=52)	30 (58)	14 (27)	10 (19)	6 (12)	22 (42)
**D. During the COVID-19 pandemic, how often did you hear about concerns from your healthcare personnel about aerosol-generating procedures (AGPs) resulting in avoidance of**					
Nebulizers (eg, preference for inhalers)	46 (87)	3 (6)	7 (13)	36 (68)	7 (13)
BiPAP, CPAP (N=52)	41 (79)	10 (19)	6 (12)	25 (48)	11 (21)
High-flow nasal cannula (N=50)	36 (72)	7 (14)	10 (20)	19 (38)	14 (28)
Intubation (N=52)	37 (71)	5 (10)	12 (23)	20 (38)	15 (29)
**E. Does your facility use intubation boxes (clear plastic box placed around patient’s head as an extra barrier against airway secretions)? (N=51)**					
Not for any patients	29 (56)				
Yes, for COVID-19 patients only	11 (22)				
Yes, universally for all patients	11 (22)				
**IF your facility uses intubation boxes, how often have you heard about the following?**					
Difficulty with intubation (eg, multiple attempts) (N=31)	15 (48)	14 (45)	1 (3)	0 (0)	16 (52)
Difficulty responding to code blue while using intubation box (N=30)	4 (13)	2 (7)	1 (3)	1 (3)	26 (87)

Note. IR, interventional radiology; TEE, transesophageal echocardiogram; EGD, esophagogastroduodenoscopy; OR, operating room; CABG, coronary artery bypass graft; ex-lap, exploratory laparotomy; PPE, personal protective equipment; IV, intravenous; BiPAP, bilevel positive airway pressure; CPAP, continuous positive airway pressure; ED, emergency department; PAPR, powered air purifying respirator.

a Percentages calculated among total respondents for each question.

b No. of hospital respondents = 53 for each question unless otherwise stated.

c “Ever” composite calculated as sum of response selections of “a few times,” “sometimes,” and “often.”

Almost all IP leaders, 51 (96%) of 53 responding facilities, received requests to increase air exchanges between patients occupying ED or operating rooms; 33 (64%) of 52 facilities received requests to change operative air pressure from positive to negative, and 30 (58%) reported requests for procedure modifications (eg, discouraging intraoperative cauterization due to aerosol concerns). Use of nonrecommended PPE affecting surgical procedure times was reported by 37 (71%) of 52 facilities, and 40 (75%) of 53 facilities reported difficulty completing a procedure due to reduced visibility through face shields or goggles.

Overall, 46 (87%) of 53 responding facilities reported clinician avoidance of both noninvasive respiratory treatments not known to have aerosol transmission risk, such as nebulizers (46 of 53, 87%) and high-flow nasal cannula (36 of 50, 72%), and avoidance of invasive respiratory procedures with known aerosolization risk such as intubation (37 of 52, 71%). On the other hand, 26 (49%) of 53 reported occurrences of early intubation (before definitive need) to reduce exposure risks through mechanical ventilation. Use of “intubation boxes” (ie, clear plastic barriers around a patient’s head to protect HCP from respiratory secretions) was reported by 22 (43%) of 51 facilities, and 11 (50%) of these 22 reported universal use for all patients regardless of COVID-19 status. Among facilities using intubation boxes, 13 (59%) of 22 reported difficulty performing intubation or code-blue procedures (4 of 22, 18%).

Respondents reported occupational health hazards of PPE overuse including facial skin irritation, dermatitis or skin breakdown due to face masks (52 of 53, 98%); carbon dioxide narcosis symptoms from N95 or double masking (29 of 53, 55%); and falling or tripping (7 of 52, 13%) (Table 2).

**Table 2.** Occupational Hazards Related to Personal Protective Equipment

How often have you heard about the following occurring in workers using personal protective equipment?	Ever, No. (%)^a,b^	A Few Times (1–2×), No. (%)^b^	Sometimes (3–5×), No. (%)^b^	Often (>5×)^b^	Never, No. (%)^b^
Facial skin irritation due to mask (contact dermatitis, skin breakdown) (N=53)	52 (98)	14 (26)	21 (40)	17 (32)	1 (1)
CO_2_ narcosis (eg, headache, lethargy, dizziness while wearing N95 or double masking) (N=53)	29 (55)	14 (26)	9 (17)	6 (11)	24 (45)
Falling or tripping while wearing multiple layers of PPE (goggles plus face shield) (N=52)	7 (13)	5 (10)	2 (4)	0 (0)	45 (86)

Note. CO_2_, carbon dioxide; N95, facepiece respirator capable of filtering at least 95% of airborne particles; PPE, personal protective equipment.

a “Ever” is the composite calculated as sum of response selections of “a few times,” “sometimes,” and “often.”

b Percentages calculated among total respondents for each question.

## Discussion

Early in the COVID-19 pandemic, HCP concerns about COVID-19 exposure resulted in broad application of overly cautious practices without differentiation between high- or low-exposure activities. Although pandemic responses necessitated changes in hospital operations (eg, cancelling nonurgent surgeries) to accommodate COVID-19 patients and HCP provided lifesaving care to innumerable patients, our results show that concerns about transmission risk added to procedure delays, cancellations, modifications, and unnecessary PPE use, adversely affecting HCP physical well-being and patient care delivery.

Procedural delays and unexpected cancellations were reported across a wide spectrum of transmission risk, including among those with minimal respiratory transmission risk. Many studies have shown the serious consequences of COVID-19–associated delays on surgical, cancer, cardiac, or diabetes care, underscoring the need to limit COVID-19 prevention practices to those that are truly necessary.^3–6^ Preprocedural testing contributed to care delays in large numbers of patients, most of whom did not have COVID-19.^7^ Although clinical circumstances can warrant delaying surgery due to COVID-19, positive tests often resulted in reflexive cancellations despite the fact that positive PCRs often indicate convalescent disease and that many surgeries can be safely performed with appropriate PPE. This strategy has remained active in many facilities despite lower frequency and severity of COVID-19 in the postvaccine era and despite highly effective IP protocols.^8^

Concerns about aerosolization were similarly pervasive and included avoidance of noninvasive respiratory treatments not known to produce infectious aerosols. Standardized definitions of AGPs that constitute true pathogen transmission risk are needed to prevent exposure concerns from driving broader definitions that could have had untoward consequences. In addition, studies demonstrating real-world effectiveness of PPE and standardized IP processes are needed so that pandemic scenarios do not potentiate unnecessary fear and actions to avoid exposures.

The use of “extra” prevention practices beyond evidence-based strategies can undermine current standards for high-quality, safe patient care and the invaluable HCP care provided in the setting of a pandemic.^9,10^ Our findings of clinical practice modifications suggested that HCP concern about COVID-19 exposure superseded adherence to well-vetted clinical and IP guidelines. We also found that extra PPE layers compromised HCP visibility, mobility, and function with unintended effects on both patient care and HCP health. Investments in HCP education on IP concepts to reduce harms that can inadvertently arise from overuse of precautions are needed.

This study had several limitations. The survey design captured anecdotal experience from a convenience sample of US hospital IP leaders. We did not assess the persistence of these early pandemic experiences, and emotional drivers of such experiences were inferred.

In summary, we found multiple examples of HCP modifying PPE and clinical practices with detrimental effects on patients and HCP across many hospitals, likely driven by excessive caution to avoid exposures. These findings have important implications for pandemic planning and response, including the need to emphasize balanced approaches to precautions, to prioritize HCP educational campaigns to manage safety concerns, and to generate an evidence base that can guide appropriate IP practices.

## Supplementary material

For supplementary material accompanying this paper visit https://doi.org/10.1017/ice.2023.89. click here to view supplementary material
