# Supplementary material for: Coronavirus disease 2019 (COVID-19) infection prevention practices that exceed Centers for Disease Control and Prevention (CDC) guidance: Balancing extra caution against impediments to care
Source: Infect Control Hosp Epidemiol. 2023 Jun 1;44(12):2074–7. doi: 10.1017/ice.2023.89 (PMC10755143; doi:10.1017/ice.2023.89)
Supplement: Supplementary file 1 [file S0899823X23000892sup001.docx]

**Facility Survey – Unintended Consequences of “Abundance of Caution” Rationale for COVID Exposure on Clinical Decision Making**

**Objectives** – (1) To capture early facility experiences with balancing healthcare worker (HCW) concerns about COVID exposure and delivery of timely, high quality patient care for both COVID and non-COVID patients. (2) To highlight key areas of research and/or educational efforts to address HCW concerns and optimize patient care in the setting of a pandemic.

**Survey Directions:** Please fill out the below survey that evaluates experiences in usual patient care activities during the early phases (e.g., February-April, 2020) of the COVID pandemic affecting both COVID and non-COVID patients. All responses will be kept confidential and will be aggregated across facilities. Individual responses will not be published.

**Intended Survey Respondent:** Hospital epidemiologist, infection prevention leadership or team member.

**The survey is estimated to take approximately 10-15 minutes to complete.**

**Facility name not needed; a de-identified code name will be assigned upon receipt.**

| - - 1. **Facility Characteristics:** |  |  |  |
| --- | --- | --- | --- |
| Number of acute care beds | <200 | 200-400 | >400 |
| Number of ICU beds | <30 | 30-60 | >60 |
| Does your facility provide Level 1 trauma care? | Yes | No | Unsure |
| Which of the following best describes your facility?  Academic medical center  Large Community hospital with graduate medical education training (GME) program  Large Community hospital without GME program  Small Community hospital with graduate medical education training (GME) program  Small Community hospital without GME program | | | |
| Does your facility provide services for highly immunocompromising conditions (e.g., bone marrow transplantation, induction chemotherapy, etc.) | Yes | No | Unsure |
| Has your facility experienced a peak in COVID patients? | Yes | No | Unsure |
| If yes, approximately when did your facility peak cases? | **Month**: Feb Mar Apr May  **Week of month chosen above**: 1^st^ 2^nd^ 3^rd^ 4^th^ | | |

**Impact of COVID Concerns on Usual Patient Care**

| - - 1. **During the COVID pandemic, how often did you hear about concerns from your healthcare workers about aerosol-generating procedures resulting in avoidance of:** | **Never** | **A few times (1-2x)** | **Sometimes (3-5x)** | **Often (>5x)** | **Comments:** |
| --- | --- | --- | --- | --- | --- |
| Intubation |  |  |  |  |  |
| Non-invasive positive pressure ventilation (BiPAP, CPAP) |  |  |  |  |  |
| Nebulizers (e.g., preference for inhalers) |  |  |  |  |  |
| High flow nasal cannula |  |  |  |  |  |
| Others (please specifiy): | | | | | |
| - - 1. **During the COVID pandemic, how often did you hear about the following occurring due to healthcare worker concerns about COVID transmission:** | **Never** | **A few times (1-2x)** | **Sometimes (3-5x)** | **Often (>5x)** |  |
| Early intubation instead of attempting high flow nasal cannula or other non-invasive positive pressure ventilation |  |  |  |  |  |
| Unexpected cancellation/delay of a surgical procedure requiring general anesthesia (e.g., CABG, vascular surgery, biopsy, ex-lap) |  |  |  |  |  |
| Unexpected cancellation/delay of a non-OR procedure (bronchoscopy, ENT, IR, cardiac catheterization, TEE, endoscopic GI procedures) |  |  |  |  |  |
| Delay of procedures due to request for pre-procedural testing to “rule out” COVID |  |  |  |  |  |
| **If able, please describe any specific scenarios or other types of procedures impacted by COVID:** | | | | | |
| - - 1. **How often have you heard about patients with chronic diseases requiring an ED visit due to disruption in routine medical management (e.g, worsening diabetes or hypertensive urgency, high-risk prenatal visits, seizures)?** |  |  |  |  |  |
| **If able, please describe the most common ambulatory conditions impacted by COVID at your facility**. | | | | | |
|  | **Never** | **A few times (1-2x)** | **Sometimes (3-5x)** | **Often (>5x)** | **Comments:** |
| - - 1. **How often have you heard about delays or changes in hospital care leading to longer hospital stays?** |  |  |  |  |  |
| - - 1. **How often did you encounter modifications in usual clinical workflows due to concerns about COVID transmission?** |  |  |  |  |  |
| Request to allow time for air exchanges between patients (e.g., in operating room (OR), between trauma patients in ED) |  |  |  |  |  |
| Requests for or inquiries into changing OR air pressure from positive to negative pressure |  |  |  |  |  |
| Use of extra PPE affecting surgical procedure times (e.g., added PPE (e.g. body suits, PAPRs) requiring extra time for doffing and donning) |  |  |  |  |  |
| Procedure modifications (e.g., Bovie cauterization not allowed/discouraged due to AGP concern) |  |  |  |  |  |
| Difficulty with a procedure due to double-gloving (e.g., IV insertion, central line insertion, etc.) |  |  |  |  |  |
| Delay in pre-operative nasal decolonization due to concerns about interference with pre-operative COVID screening. |  |  |  |  |  |
| **If able, please describe any other specific modifications in usual clinical care or workflows that have been impacted by concerns about COVID transmission?** | | | | | |
| - - 1. **How often have you heard about the following occurring in workers using personal protective equipment?** | **Never** | **A few times (1-2x)** | **Sometimes (3-5x)** | **Often (>5x)** | **Comments:** |
| Facial skin irritation due to mask resulting in contact dermatitis or skin breakdown |  |  |  |  |  |
| Carbon dioxide narcosis (e.g., headache, lethargy, dizziness while wearing N95 or while double masking) |  |  |  |  |  |
| Falling or tripping while wearing multiple layers of PPE (goggles plus face shield) |  |  |  |  |  |
| Difficulty completing a procedure due to: | **Never** | **A few times (1-2x)** | **Sometimes (3-5x)** | **Often (>5x)** | **Comments:** |
| Reduced visibility through face shields or goggles |  |  |  |  |  |
| Double gloving |  |  |  |  |  |
| - - 1. **Does your facility use intubation boxes (clear plastic box placed around patient’s head as an extra barrier against airway secretions)?**   No, our facility does not use intubation boxes  Yes, for COVID positive patient intubations only  Yes, for all patient intubations regardless of COVID status | | | | | |
| - - 1. **IF your facility uses intubation boxes, how often have you heard about the following?** |  |  |  |  |  |
| Difficulty with intubation (e.g., requiring multiple attempts) |  |  |  |  |  |
| Difficulty responding to code blue while using intubation box |  |  |  |  |  |
